# Commentary on human pluripotent stem cell-based blood–brain barrier models

**DOI:** 10.1186/s12987-020-00222-3

**Published:** 2020-10-19

**Authors:** Ethan S. Lippmann, Samira M. Azarin, Sean P. Palecek, Eric V. Shusta

**Affiliations:** 1 Department of Chemical and Biological Engineering, University of Wisconsin–Madison, Madison, WI USA; 2 Department of Neurological Surgery, University of Wisconsin–Madison, Madison, WI USA; 3 Present Address: Department of Chemical and Biomolecular Engineering, Vanderbilt University, Nashville, TN USA; 4 Present Address: Department of Chemical Engineering and Materials Science, University of Minnesota, Minneapolis, MN USA

**Keywords:** Blood–brain barrier, Human pluripotent stem cell, In vitro model

## Abstract

In 2012, we provided the first published evidence that human pluripotent stem cells could be differentiated to cells exhibiting markers and phenotypes characteristic of the blood–brain barrier (BBB). In the ensuing years, the initial protocols have been refined, and the research community has identified both positive and negative attributes of this stem cell-based BBB model system. Here, we give our perspective on the current status of these models and their use in the BBB community, as well as highlight key attributes that would benefit from improvement moving forward.

## Background

In vitro blood–brain barrier (BBB) models are useful for advancing understanding of BBB development and function. They can also be used to model BBB dysfunction in disease and leveraged in the screening and evaluation of new therapeutic interventions. A number of years ago, we sought to develop a BBB model derived from human pluripotent stem cell (hPSC) sources to address several key challenges in the BBB modeling field. For translational research, it is desirable to have human BBB models to bridge the gap between animal studies and human treatment. In particular, it was widely recognized that BBB models containing primary animal-sourced brain microvascular endothelial cells (BMECs) were often unable to predict human BBB properties, in part as a result of interspecies differences. Unfortunately, primary human BMEC sources are scarce, as they are isolated from brain specimens originating from autopsy or surgical resection, and cannot be significantly expanded in culture. Immortalized human BMEC lines were also in routine use, but they exhibited subpar barrier properties. It was therefore difficult to recapitulate key functional attributes of the human BBB, including passive and active barrier properties, within these models. To address these needs, we first devised a protocol whereby hPSCs, both human embryonic stem cells (hESCs) and human induced pluripotent stem cells (hiPSCs), were differentiated into a mixture of cells expressing neural and endothelial markers, followed by purification of the putative endothelial cells through selective adhesion to an extracellular matrix coating. The purified cells expressed both endothelial and BBB markers in addition to exhibiting key BBB phenotypes, including elevated transendothelial electrical resistance (TEER) as a result of robust tight junctions, representative permeability to small molecules, and polarized efflux transporter activity [1]. We defined this cell type as hPSC-derived BMECs. The resultant cells have proven useful for examining interactions between cells of the neurovascular unit [1–10], evaluating experimental drug permeability at the BBB [1, 11–16], and modeling human genetic disease using hiPSCs derived from patient sources [9, 10, 13], among other applications.

Since the initial protocol was published, several derivative protocols have been put forth by us and others. The first major protocol adaptation occurred in our follow-up publication, which described the inclusion of retinoic acid (RA) leading to substantially elevated TEER and reinforcement of VE-cadherin expression during the differentiation process [5]. Protocols have been further refined for accelerated differentiation [17] and the use of chemically-defined serum-free medium [18]. Low osmolarity medium during the initial differentiation phase has been used to obviate the need for cell purification by passaging [7], and hypoxia has also been applied during differentiation to better mimic developmental oxygen tension [19]. Finally, we described the first directed differentiation strategy that, instead of utilizing a “co-differentiation” approach with mixtures of neural and endothelial cells, transitioned differentiating hPSCs through a mesodermal intermediate, followed by endothelial specification and induction of BBB functionality [6]. One interesting observation has been that, despite the myriad of protocols now available, the majority of published studies report commonalities in endothelial marker expression and BBB function of the hPSC-derived BMECs. Furthermore, despite instances where the hPSC-derived BMECs were differentiated through vastly different methods (for example, the co-differentiation process [1] versus a transition through a mesodermal progenitor state [6]), RNA sequencing techniques indicate that these cells share similar global transcriptional profiles [6]. However, transcriptomic analyses have also revealed an unexpected feature of these BMECs in that they express a substantial number of epithelial-associated transcripts [9, 20, 21]. Given that this particular issue is of significant interest to the BBB community, we detail the endothelial and epithelial attributes of hPSC-derived BMECs and provide our perspective on current strengths and weaknesses that should be considered when deploying hPSC-derived BMECs in a research setting, and areas that need to be improved with further model refinement.

## Main text

**Fig. 1:**
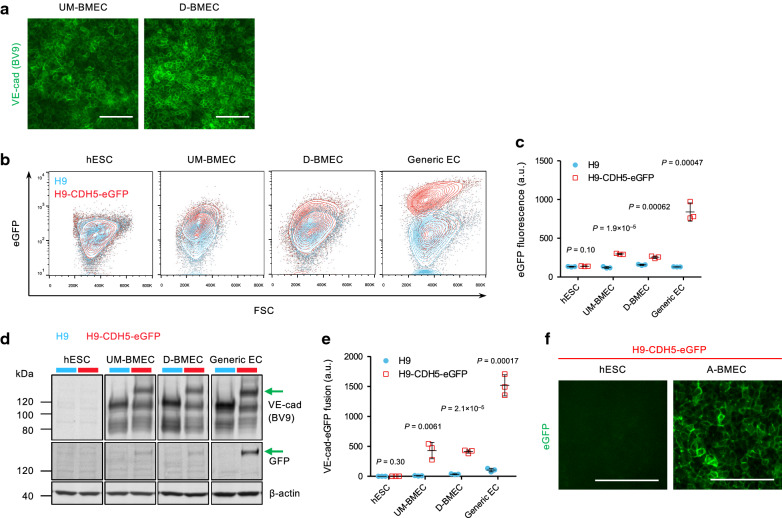
VE-Cadherin expression in H9 and H9-CDH5-eGFP hESC lines. **a** VE-cadherin immunocytochemistry of BMEC-like cells differentiated from the H9-CDH5-eGFP reporter using the RA-enhanced differentiation protocol [5] (UM-BMEC) and the chemically-defined, directed differentiation protocol [6] (D-BMEC). Scale bars: 100 μm. **b** Flow cytometry analysis of eGFP fluorescence in undifferentiated hESCs, UM-BMECs, D-BMECs, and generic hPSC-derived ECs [33] (Generic EC). Representative contour plots from biological triplicates showing eGFP expression and forward scatter (FSC) in the H9 hESC line (blue) and H9-CDH5-eGFP hESC line (red). Example gating strategy is shown in Additional file 1: Figure S1. **c** Quantification of eGFP geometric mean fluorescence intensity from flow cytometry analysis of biological triplicates of cells as described in **b**. Data are plotted as mean ± s.d. P-values: Student’s unpaired t-test. **d** Western blot for VE-cadherin, GFP, and β-actin expression in hESCs, UM-BMECs, D-BMECs, and generic ECs. Bands shown are from representative biological triplicates from the H9 hESC line (blue) and H9-CDH5-eGFP hESC line (red). Full western blots are shown in Additional file 1: Figure S2. Green arrows indicate the VE-cadherin-eGFP fusion protein bands. **e** Quantification of VE-cadherin-eGFP fusion protein abundance from anti-VE-cadherin Western blot analysis for samples as described in **d**. Data from three biological triplicates from a representative differentiation are shown. p-values: Student’s unpaired t-test. **f** eGFP fluorescence of undifferentiated H9-CDH5-eGFP hESCs and BMEC-like cells differentiated using the accelerated, chemically-defined serum free differentiation protocol [18] (A-BMEC). Scale bars: 100 μm

First, hPSC-derived BMECs possess vascular character. We and others observe PECAM-1 [1, 2, 5–7, 13, 14, 17, 19, 20, 22, 23] and VE-cadherin [1, 2, 5–7, 14, 17, 22, 24, 25] protein expression by a combination of flow cytometry, western blotting, and immunofluorescence. In addition, expression of other endothelial-associated proteins such as von Willebrand factor [1, 2, 6, 7, 20, 22], VEGFR2 [6], TIE2 [2], and SOX17 [7] has been reported. To further validate endothelial marker expression, and to avoid any potential specificity-related complications with antibody-based detection methods, we have now also used a gene-edited hESC line to illustrate VE-cadherin expression in hPSC-derived BMECs. Previously, we and others demonstrated a clear population of VE-cadherin positive cells (80–100%) by immunofluorescence and flow cytometry using anti-VE-cadherin antibodies [2, 5–7, 17, 24, 25] (e.g. Fig. 1a). Here, an H9 hESC VE-cadherin-eGFP reporter line [26] was differentiated to BMECs using three different protocols developed by our laboratories: the most widely used RA-enhanced protocol [5], the chemically-defined directed differentiation protocol that proceeds through a mesoderm lineage [6], and the accelerated, chemically-defined serum-free co-differentiation protocol based on the original RA-enhanced methods [17, 18]. The resultant BMECs were then assessed for reporter expression using a variety of techniques. eGFP fluorescence was clearly evident in the hESC-derived BMECs compared to cell-type matched controls differentiated from the parental H9 hESC line (Fig. 1b and c), albeit at lower levels than generic hESC-derived endothelial cells (ECs). Western blotting also indicated the production of VE-cadherin-eGFP fusion protein only in reporter line samples that had undergone differentiation (Fig. 1d and e). In addition, the fusion protein could be visualized at cell–cell junctions in hESC-derived BMECs but not undifferentiated hESCs (Fig. 1f). These data further demonstrate the expression of VE-cadherin in hPSC-derived BMECs and support the many prior reports of expression of endothelial proteins in hPSC-derived BMECs. These data are also consistent with reports of reduced endothelial character at both the transcript and protein levels in hPSC-derived BMECs, with lower expression levels compared to primary BMECs or generic hPSC-derived ECs [6, 20]. In the future, similar approaches using other hPSC reporter lines (e.g. PECAM-1) or proteomics analyses could be used to more comprehensively characterize BMEC marker expression and endothelial identity.

As more laboratories have tested these differentiation procedures, it has also become clear that detailed protocol refinements such as hPSC seeding density [6, 25] and line-to-line optimization of differentiation timing [1, 27], key variables for hPSC differentiation to many cellular products, are also important for successful hPSC-derived BMEC differentiation and induction of endothelial marker expression. Given our own experience and feedback we have received from other independent laboratories, it is possible to generate hPSC-derived cells having barrier properties in the absence of endothelial protein expression or with cells lacking proper endothelial protein localization under conditions of suboptimal differentiation [6]. Thus, in addition to measuring barrier formation, it is critical to ensure that hPSC-derived BMECs also express requisite endothelial markers (e.g. PECAM-1, VE-Cadherin, vWF) at the protein level, with proper subcellular localization, to confirm that the differentiation has proceeded successfully.

In addition to vascular marker expression, hPSC-derived BMECs have been shown to exhibit functional attributes expected of ECs. We have observed VEGF-dependent network formation in hPSC-derived BMECs using in vitro Matrigel assays [1], and VEGF has been shown to regulate *PLVAP* expression in a three-dimensional BBB model that contains hPSC-derived BMECs [10]. hPSC-derived BMECs also respond to shear; we have observed sprouting-like behavior after culturing these cells in engineered hydrogel matrices under constant perfusion [28], and others have observed similar phenotypes in hydrogels in response to growth factors and other stimuli such as oxidative stress [29]. In addition, application of shear after differentiation also led to activation of cholesterol metabolism, proliferation, and angiogenesis transcriptional pathways compared to static controls [9]. Furthermore, hPSC-derived BMECs respond to some inflammatory mediators. For instance, we have demonstrated that administration of the inflammatory cytokine TNFα can upregulate ICAM-1 expression in hPSC-derived BMECs [6], and others have demonstrated that TNFα upregulated both ICAM-1 and VCAM-1 leading to increased adhesion of peripheral blood mononuclear cells [30], while barrier properties decreased after exposure to TNFα, IL-8, and IL-1β [9]. Although the hPSC-derived BMECs respond to inflammatory conditions, the complete characterization of hPSC-derived BMEC immunophenotype remains to be evaluated. Taken together, a body of evidence from multiple independent researchers indicates hPSC-derived BMECs express vascular markers and exhibit a range of vascular phenotypes.

However, as noted above, advances in transcriptomics, including bulk RNA sequencing (RNAseq) and single cell RNA sequencing (scRNAseq), have identified an underlying epithelial gene expression signature in hPSC-derived BMECs. We first noted epithelial protein expression in a previous study where we demonstrated a selective upregulation of VE-cadherin in the BMECs upon RA treatment, whereas E-cadherin was basally expressed in the final BMEC product but not induced by RA [5]. In 2018, Delsing and colleagues more deeply described this epithelial signature by RNAseq comparison of two hiPSC-based BBB models that used different sources of endothelium [20]: generic hiPSC-derived ECs and hiPSC-derived BMECs generated using the RA-enhanced differentiation protocol [5]. Specifically, they noted an epithelial transcriptional signature and depressed, though detectable, expression of endothelial genes in hiPSC-derived BMECs compared with the generic hiPSC-derived EC model. Vatine and colleagues more recently used RNAseq to interrogate hiPSC-derived BMECs and also noted expression of both epithelial and endothelial transcripts [9]. Finally, Lu and colleagues deployed both RNAseq and scRNAseq techniques to further demonstrate epithelial transcript expression in hPSC-derived BMECs [21]. Thus, in light of this epithelial gene expression profile described by multiple groups, we suggest these cells should now be more appropriately referred to as hPSC-derived BMEC-*like* cells.

Despite this mixed endothelial-epithelial transcriptional profile, our perspective is that for the majority of in vitro studies, the predictive capacity of human BBB function is the most important characteristic in selecting and deploying a BBB model. For example, to complement their transcriptomics analyses, Delsing and colleagues performed an evaluation of BBB properties. Despite their more representative vascular character, BBB models based on generic hiPSC-derived ECs did not recapitulate BBB function nearly as well as hiPSC-derived BMEC-like cells in terms of passive barrier, efflux transport, and drug permeability phenotypes [20]. Moreover, transcriptomic analyses indicate that there are global similarities between hPSC-derived BMEC-like cells generated by several research groups using a variety of protocols [6, 21]. However, by relying solely on transcriptomic analysis, it is difficult to distinguish differences in BBB phenotypes or cellular identities that arise from different differentiation trajectories. For example, addition of RA to the original differentiation protocol drastically increased TEER to near-physiologic levels via restructuring of tight junctions rather than through changes in tight junction protein expression levels [5, 24], a feature that would potentially have been overlooked by profiling tight junction transcripts alone. Further, our directed differentiation protocol guides hPSCs to BMEC-like cells in a developmentally-relevant progression through mesodermal and endothelial progenitor lineages, while yielding a final BMEC-like population that was very similar transcriptomically to the earlier approaches relying on co-differentiating neural and endothelial populations [6]. Thus, while -omics analyses are valuable tools for profiling cell states, it is also important to probe cell structure and function, particularly in assessing suitability of a cell type for in vitro modeling applications.

Collectively, current literature suggests that hPSC-derived BMEC-like cells exhibit both endothelial and epithelial character. As a result, practitioners of BBB models should exercise care in employing hPSC-derived BMEC-like cells since they are not identical to human BMECs in vivo and therefore may not be appropriate for all applications. However, like all models, applications must be carefully matched to the model capability, and model predications should be validated and tested in complementary in vitro and in vivo assays. Importantly, hPSC-derived BMEC-like cells are currently the only available BBB model possessing both high passive barrier and functional transporter characteristics that can serve as a reasonable facsimile of the in vivo human BBB [6, 7, 9, 13, 14]. For instance, permeability of drugs and nutrients across a monolayer of hPSC-derived BMEC-like cells correlated with in vivo rodent brain transport rates [1], and subsequently, PET radioligand transport across BMEC-like cells correlated with human BBB permeability [16]. It has also been demonstrated that these cells can be used to compare the relative in vitro BBB permeability of known drugs, to evaluate the permeability and efflux profile of experimental drugs, and to explore the mechanisms of nutrient transport [1, 11–16]. hPSC-derived models can also be used to compare transport of antibodies across the BBB to identify antibody- and species-dependent effects on trans-BBB transport [7, 19] and identify new antibodies capable of binding the human BBB [31]. In the case of human genetic disease, hPSC-derived BMEC-like cells could play a unique role in modeling human disease when animal models are lacking. For instance, hiPSC-derived BMEC-like cells can be an invaluable tool to examine molecular transport at the human BBB using patient-sourced, diseased hiPSC lines [9, 13], and to examine the impact of genetic risk factors on disease modifying processes such as neurovascular amyloid deposition [10]. In addition, hPSC-derived BMEC-like cells respond to cues originating from co-cultured neurovascular unit cells such as astrocytes, pericytes, and neurons [1–9]. Importantly, we have shown that hPSC-derived BMEC-like cells exhibit barrier tightening in response to astrocyte and neuron cues as a result of improved tight junction localization, which mimicked the effects observed in primary BMECs [4]. Moreover, hiPSC-derived BMEC-like cells exhibited both barrier tightening and a reduction of non-specific transcytosis when exposed to brain pericyte-derived cues, and again these induced properties mimicked those seen in primary BMECs [8]. Therefore, hPSC-derived BMEC-like cells have served as an enabling platform to successfully model aspects of BBB barrier modulation, molecular transport, and neurovascular response.

Still, it is crucial to note that hPSC-derived BMEC-like cells have remaining challenges that need to be addressed. Most relevant to this commentary, induction and maintenance of endothelial character with a corresponding reduction of epithelial character continues to be a goal. Factors such as hypoxia [19], shear stress [9, 19], and three dimensional architecture [10, 28, 29] have been suggested to increase vascular character, and other models based on induction of BBB character in generic hiPSC-derived ECs are beginning to emerge [32]. Despite these advances, we believe it is prudent to exercise caution when utilizing hPSC-derived BMEC-like cells for studies where the endothelial phenotype is crucial. For instance, hPSC-derived BMEC-like cells have not yet been fully characterized for their immune cell adhesion repertoire and capability for supporting immune cell migration. Also, given the depressed endothelial transcript profile, care should be taken when using hPSC-derived BMEC-like cells for modeling BBB development. As mentioned earlier, it is important to complement such studies with in vivo models and human tissue to provide confidence in the accuracy of outcomes. As one example, the workflow employed by Tsai and colleagues to study neurovascular cell-specific deficiencies imparted by APOE status combined hPSC-derived BMEC-like cells and human data [10]. In another example, antibodies that bind hPSC-derived BMEC-like cells were validated by assessing vascular targeting in human brain tissue sections [31]. Thus, hPSC-derived BMEC-like cells can be a valuable complementary tool in BBB research.

## Conclusions

In conclusion, based on their recapitulation of BBB phenotypes, hPSC-derived BMEC-like cells are frequently used as in vitro models of the BBB. However, caution must be taken in employing these cells as BBB models since they also express epithelial genes and proteins. Thus, it is important to employ hPSC-derived BMEC-like cells for particular applications to which they are suited and to validate results from these models using complementary models and experimental techniques. The use of hPSC-derived cells in modeling the BBB continues to evolve with the development and application of new differentiation and characterization protocols. We fully expect that improvements will continue to be made to generate hPSC-derived BMEC-like cells that more faithfully model the BBB.

## Supplementary information

**Additional file 1**. Additional figures and tables.

## Data Availability

All data reported in this study are included in this published article and its additional information files.

## Abbreviations

BBB: Blood–brain barrier
hPSC: Human pluripotent stem cell
hESC: Human embryonic stem cell
hiPSC: Human induced pluripotent stem cell
EC: Endothelial cell
BMEC: Brain microvascular endothelial cell
TEER: Transendothelial electrical resistance
