# Supplementary material for: Commentary on human pluripotent stem cell-based blood–brain barrier models
Source: Fluids Barriers CNS. 2020 Oct 19;17:64. doi: 10.1186/s12987-020-00222-3 (PMC7574179; doi:10.1186/s12987-020-00222-3)

Additional Methods

hPSC maintenance and differentiation

H9 and H9-CDH5-eGFP hESCs [26] were maintained on Matrigel (Corning) in TeSR-E8 medium (Stemcell Technologies). UM-BMECs were differentiated as previously described [5] with the modification that 1× B-27 Supplement (Life Technologies) was substituted for platelet-poor plasma-derived serum (PDS) in the endothelial medium used from day 6 to day 8 of differentiation. D-BMECs were differentiated as previously described [6]. Both UM-BMECs and D-BMECs were evaluated at D8. H9-CDH5-eGFP hESCs used in A-BMEC experiments were maintained on Matrigel (Corning) in E8 medium prepared as previously described [17]. A-BMECs were differentiated as previously described [18] in MatTek 35 mm glass bottom dishes and evaluated at D6. Generic ECs were differentiated as previously described [33] with the modification that endothelial progenitors were sorted at D5 using CD31 magnetic microbeads (Miltenyi Biotec) and subsequently cultured in Human Endothelial Serum Free Medium (Life Technologies) supplemented with 1× B-27 Supplement and 10 ng/mL FGF2 (Waisman Biomanufacturing), and generic ECs were analyzed on D8.

Immunocytochemistry

Cells were fixed for 10 min with cold 100% methanol and washed 3× in Dulbecco's PBS without calcium or magnesium (DPBS). Cells were blocked with DPBS containing 10% goat serum for 1 h at room temperature with gentle shaking. Cells were incubated with mouse anti-VE-cadherin (BV9, Santa Cruz Biotechnology) antibody diluted 1:25 in DPBS containing 10% goat serum overnight at 4°C with gentle shaking. Cells were washed 3× with DPBS and incubated with goat anti-mouse IgG-AlexaFluor 647 (Life Technologies) diluted 1:200 in DPBS containing 10% goat

serum for 1 h at room temperature with gentle shaking. Cells were washed 3× with DPBS and imaged with a Nikon Eclipse Ti2-E microscope.

Visualization of eGFP expression by fluorescence microscopy

Undifferentiated H9-CDH5-eGFP hESCs and D6 A-BMECs were rinsed 2× with DPBS and fixed for 20 min in 4% paraformaldehyde. Cells were washed 3× in DPBS, 5 min per wash, and eGFP fluorescence was imaged with a Leica DMi8 microscope. Cells were not incubated with any primary or secondary antibodies prior to imaging.

Flow cytometry

Cells were dissociated with Accutase (Innovative Cell Technologies) at 37°C, passed through 40 µm filters into 4× volume of DMEM/F-12 (Life Technologies), and centrifuged at 200×g for 5 min. Cell pellets were resuspended in cold DPBS containing 0.5% BSA, 2 mM EDTA, and 2 µg/mL DAPI and immediately analyzed on an Attune NxT flow cytometer (ThermoFisher). Debris were removed using side scatter (SSC)-forward scatter (FSC) gating, single cells selected using FSC-H versus FSC-A and SSC-H versus SSC-A, and live cells selected using a DAPI-negative gate. Example flow cytometry plots depicting this gating strategy are shown in Supplementary Figure S1.

Western blotting

Cells were lysed with cold RIPA lysis buffer (Rockland) supplemented with Halt protease inhibitor cocktail (Thermo Scientific). A BCA assay was used to determine protein concentration, and equal amounts of protein were resolved on 4–12% tris-glycine gels. Proteins were transferred to nitrocellulose membranes and blocked in tris-buffered saline containing 0.1% Tween-20 (TBST) and 5% nonfat dry milk for 1 h at room temperature. Membranes were incubated with primary antibodies diluted in TBST containing 5% milk overnight at 4°C: Membrane 1: mouse anti-VE-

cadherin (BV9, Santa Cruz Biotechnology) and rabbit anti-β-actin (13E5, Cell Signaling Technology); Membrane 2: mouse anti-GFP (B-2, Santa Cruz Biotechnology). Membranes were washed 5× with TBST and incubated with secondary antibodies diluted in TBST containing 5% milk for 1 h at room temperature: Membrane 1: goat anti-mouse IgG IRDye 800 (LI-COR) and goat anti-rabbit IgG IRDye 680 (LI-COR); Membrane 2: goat anti-mouse IgG IRDye 800 (LI-COR). Membranes were washed 5× with TBST and imaged using a LI-COR Odyssey.

Statistics

All experiments were conducted in biological triplicate (e.g., three independently differentiated wells of a 6-well plate). Data shown are from representative differentiations of the H9 and H9-CDH5-eGFP hESC lines; all experiments were replicated in an additional differentiation of each line. Student's unpaired t-test was used for all statistical analyses.

Figure S1: Example flow cytometry gating strategy for data shown in Fig 1b.

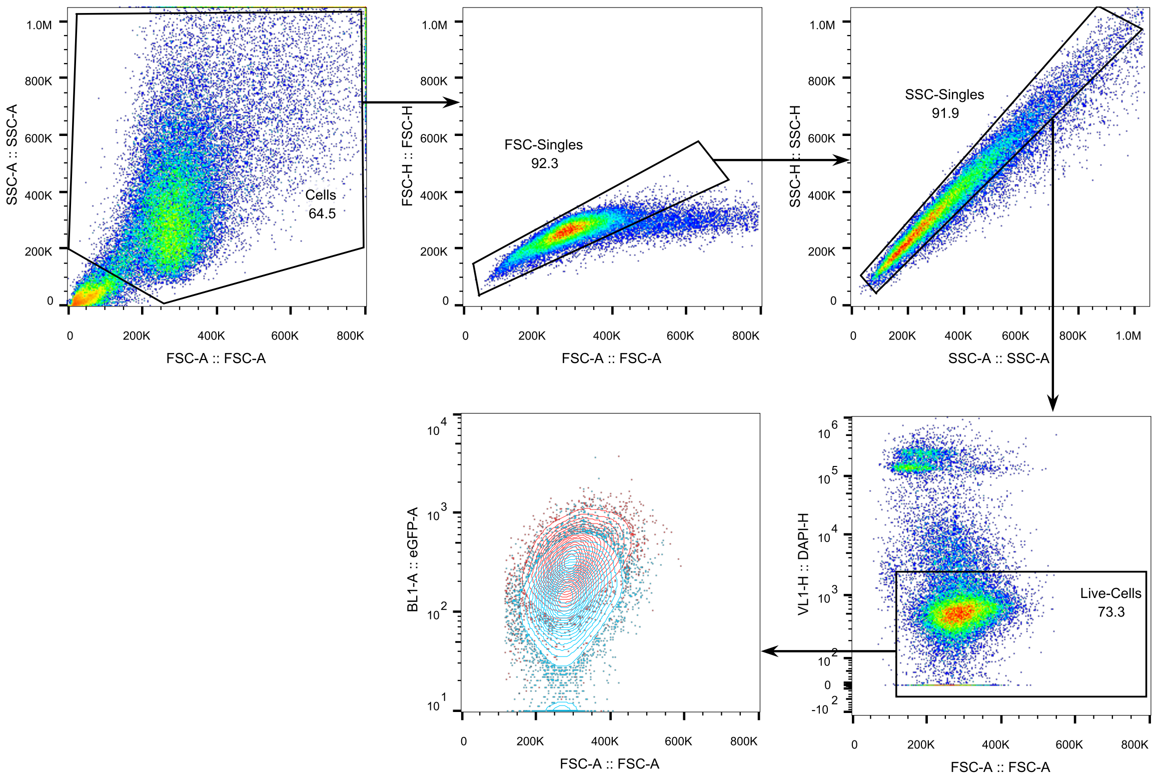

Figure S2: Uncropped Western blot images. Dashed lines indicate the bands displayed in Figure 1d.

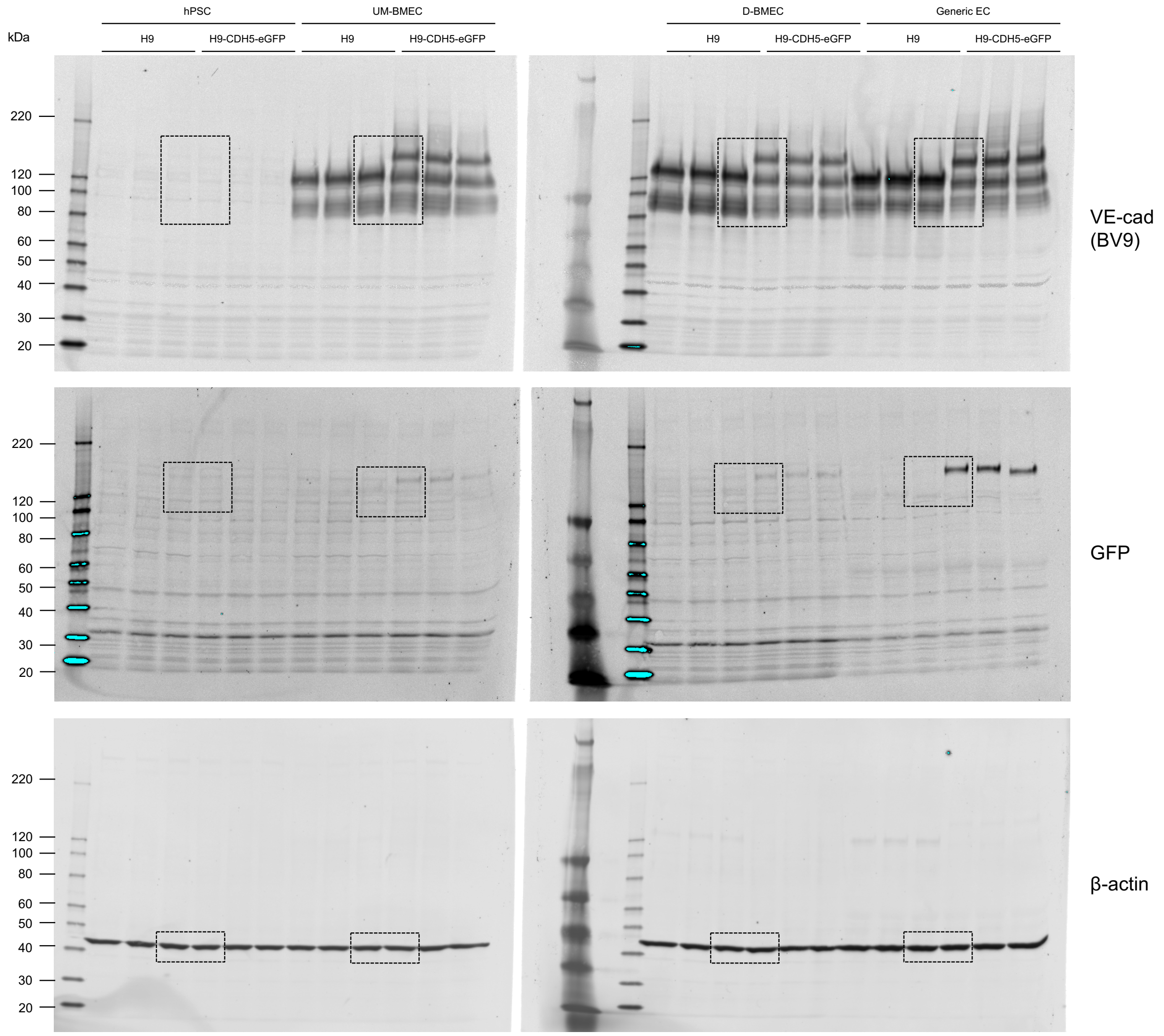

Supplement: Supplementary file 1 — Additional file 1. Additional figures and tables. [file 12987_2020_222_MOESM1_ESM.pdf]
